# Nonmotor symptoms in spinocerebellar ataxias (SCAs)

**DOI:** 10.1186/s40673-019-0106-5

**Published:** 2019-08-27

**Authors:** Adriana Moro, Mariana Moscovich, Marina Farah, Carlos Henrique F. Camargo, Hélio A. G. Teive, Renato P. Munhoz

**Affiliations:** 1 Movement Disorders Unit, Neurology Service, Internal Medicine Department, Hospital de Clínicas, Federal University of Paraná, 50 Teixeira Soares Street, Batel, Curitiba, PR CEP 80240-440 Brazil; 2 Department of Medicine, Pequeno Príncipe College, Curitiba, PR Brazil; 3 Department of Neurology, Universitätsklinikum Schleswig-Holstein, Kiel, Germany; 4 Neurology Service, Hospital Universitário Cajurú, Catholic University of Paraná, Curitiba, PR Brazil; 5 Neurological Diseases Group, Graduate Program of Internal Medicine, Internal Medicine Department, Hospital de Clínicas, Federal University of Paraná, Curitiba, PR Brazil; 6 Department of Medicine, Morton and Gloria Shulman Movement Disorders Centre, Toronto Western Hospital, University of Toronto, Toronto, ON Canada

**Keywords:** Nonmotor symptoms, Spinocerebellar ataxia, SCA

## Abstract

Nonmotor symptoms (NMS) have been increasingly recognized in a number of neurodegenerative diseases with a burden of disability that parallels or even surpasses that induced by motor symptoms. As NMS have often been poorly recognized and inadequately treated, much of the most recent developments in the investigation of these disorders has focused on the recognition and quantification of NMS, which will form the basis of improved clinical care for these complex cases. NMS have been only sparsely investigated in a limited number of spinocerebellar ataxias (SCAs), particularly SCA3, and have not been systematically reviewed for other forms of SCAs. The aim of the present study was to review the available literature on the presence of NMS among different types of SCAs.

## Introduction

Spinocerebellar ataxias (SCAs) comprise an extensive and heterogeneous group of neurodegenerative diseases with autosomal dominant inheritance [1]. Most epidemiological studies reveal an overall prevalence of SCAs falling within 1 to 5 cases per 100,000 inhabitants worldwide. For instance, an epidemiological study from the Netherlands with 391 genetically tested individuals found a prevalence of 3 cases per 100,000 inhabitants [2], similar to another Norwegian study showing a prevalence of 4.2/100.000 [3]. Most of the geographic and ethnic variation, however, comes from specific subtypes of SCAs, which already exceeds 30, the most common being types 1, 2, 3, 6 and 7 [4].

Despite their inherent heterogeneity, the SCAs present certain consistent characteristics, such as the obvious core features of an ataxic syndrome and its phenomenological correlates, and the pathological substrate of a degenerative process involving the cerebellum and/or its connections [1]. From a clinical standpoint, the ataxic syndrome seen in SCAs typically includes a diverse composite of clinical manifestations including the signs of axial and appendicular ataxia in the setting of signs such as gait disorders, dysmetria, dysdiadocokinesia, intention tremor, dysarthria and a variety of oculomotor findings.

Involvement of other structures of the nervous system in SCAs is frequently present, including the basal ganglia, brainstem nuclei, pyramidal tracts, posterior columns and the anterior horn of the medulla [4, 5]. Patients may present add with extracerebellar neurological manifestations, such as dementia, epilepsy, visual disturbances, peripheral neuropathy, supranuclear ophthalmoplegia, pyramidal tract signs, and movement disorders such as parkinsonism, myoclonus, chorea and dystonia [6–8]. Nonmotor symptoms (NMS) of neurodegenerative diseases are common but often not recognized in daily clinical practice not only due to the lack of spontaneous reporting by patients but also due to the absence of systematic questioning by their assisting physicians. In Parkinson’s disease (PD), for example, NMS are very prevalent throughout the course of the disease and occasionally may be prodromal features [9, 10].

The aim of this article is to review the main NMS of the SCAs.

### Nonmotor symptoms

Among the SCAs, due to the characteristic multisystemic neuropathological involvement, NMS have been increasingly studied and identified [11]. Pain, cramps, fatigue, autonomic dysfunction, sleep disorders, psychiatric symptoms, cognitive deficits, and olfactory dysfunction are some of the NMS already reported in various forms of SCAs that will be described in detail in this review. Table 1 summarizes NMS in SCAs, their prevalence, severity and specific details. Nonmotor symptoms are frequent and interfere with the quality of life of patients with spinocerebellar ataxias, in particular the presence of pain, cramps and fatigue, as well as autonomic, sleep, psychiatric, cognitive and olfactory disorders [11].

**Table 1:** Nonmotor symptoms in spinocerebellar ataxias^a^

	SCA1	SCA2	SCA3	SCA6	SCA7	SCA10
Fatigue [12–15]						
Severity	–	–	+++	++	–	+
Prevalence	NA	NA	36%	21%	NA	24%
Pain [15–17]						
Severity	NA	NA	++	NA	NA	–
Prevalence	NA	NA	36–47%	NA	NA	18%
Dysautonomia [15, 18–25]						
Severity	++	++	++	–	NA	–
Prevalence	67–70%^b^	20–95%^b^	22–100%^b^	0%	NA	1–8%
RBD [15, 26, 27]						
Severity	NA	–	++	–	NA	+
Prevalence	NA	NA	43–55%	NA	NA	10–20%
Depression [15, 28–33]						
Severity	+	+	+/2	+/2	NA	+
Prevalence	25–50%	23–33%	13–60%	27–75%	NA	62%
Cognition [15, 34–46]						
Severity	+	+/2	+/2	+	+/3	+
Prevalence	50%	25%	33%	45%	50%	33%
Hyposmia [47–52]						
Severity	NA	++	–	NA	+/2	–
Prevalence	NA	50%	NA	NA	33%	NA

RBD: REM sleep behavior disorder; + present (severity rated as ½ very mild, 1:mild, 2: moderate, 3: severe); − no different from controls or not detected; NA: not available

^a^Table made from comparison of data found in the cited references

^b^High prevalence of asymptomatic cardiac dysatonomia

#### Fatigue

Fatigue is a prevalent symptom in neurodegenerative diseases, and, despite being commonly considered in multiple sclerosis, it is an equal source of disability in other diseases, such as PD and SCA3 [12]. More than 10 years ago, Friedman & Amick [12] reported on the frequency and severity of fatigue among 28 SCA3 cases compared to 21 healthy controls. Severe fatigue was significantly more frequent in SCA3 (61 versus 14%) with a positive correlation with daytime sleepiness scores. In addition, fatigue, not daytime sleepiness, was correlated with disease duration and need for wheelchair but not with age or gender. Another study also found an association of fatigue with mood disorders such as anxiety and major depression [53], what could be a neurovegetative symptom of depression, a common association in general.

Brusse et al. [13] studied the prevalence of fatigue in a sample of 123 patients with various forms of SCA, including, SCA1, SCA2, SCA3, SCA6, SCA7, SCA13, SCA14, SCA17 and individuals with autosomal dominant cerebellar ataxia without identified gene mutation (ADCA). SCA3 cases had the highest prevalence of fatigue, followed by cases with ADCA and SCA6. As a whole, 69% presented high fatigue scores, 70% of them considered the symptom as one of the three most disabling. The best predictors of fatigue after multivariate analysis were physical and mental functioning, sleep and depression score.

Similarly, the study by Martinez et al. [14] found higher fatigue scores among patients with SCA3 compared to controls, which, after multiple regression analysis, was significantly associated with scores for depression and excessive daytime somnolence. A study from our group, comparing patients with SCA3, SCA10 and healthy controls, analyzed fatigue separated into physical and cognitive domains found that the physical aspect was more severely affected in SCA3 and SCA10 compared to controls, more so for SCA3 [15].

#### Pain syndrome and muscle cramps

Muscle spasms or cramps are often associated with lower motoneuron disorders but have also been frequently described in SCAs, mainly those with known anterior horn cell pathology, such as SCA2 and SCA3 [54].

Kanai et al. [54], for instance, studied the neurophysiological correlates of cramps in SCA3, identifying it as severe, frequent, and disabling in 80% of their sample. This prevalence was similar to that of amyotrophic lateral sclerosis and much higher than in cases of peripheral axonal neuropathy. The study tried to shed light into the underlying pathological changes demonstrating axonal excitability significantly greater in SCA3 patients than in normal subjects, probably reflecting axonal regeneration or collateral sprouting. Another study also found cramps in more than 80% of a sample of SCA3 patients, identified by 25% of them as their first symptom. Their frequency varied from1 to 90 episodes a week and cramps were the chief complaint in almost a third of all cases. Lower extremities were more frequently affected but face and trunk were also involved [55].

These symptoms were also found in three of four affected members of a unique family with both SCA2 and SCA10 [56]. In SCA2, cramps have been detected in the early pre-symptomatic phase of the disease, along with other nonspecific signs such as sensory and sleep disorders, hyperreflexia, and autonomic symptoms [57].

The recognition of pain as an important characteristic of neurodegenerative diseases, such as PD and multiple system atrophy (MSA), has been emphasized in recent years, being present in up to half of patients [58, 59]. Among the heredodegenerative ataxias, disabling painful spasms have been described in Friedreich ataxia [60]. Among the SCAs, a large multicenter study of patient-rated symptoms found pain/discomfort in almost half of 526 patients [16]. While the presence of pain in most specific types of SCAs has not been formally studied, in SCA3 it can be present in up to 47% of the patients as shown by França et al. [17]. This study also showed that in a significant proportion of this population can be continuous, may precede any symptom of ataxia, commonly has musculoskeletal characteristics, does not correlate with disease duration, gender, or ataxia severity of ataxia, but does so for the size of expanded CAG repeats. Similar findings were described in another study comparing SCA3 and SCA10, where only those with SCA3 had a higher incidence of chronic pain compared to controls [15].

#### Autonomic symptoms

Autonomic dysfunctions including symptomatic orthostatic hypotension, constipation, urinary and erectile dysfunctions are prominent features seen in specific neurodegenerative diseases such as early MSA, Lewy body dementia (LBD) and advanced PD [61, 62]. The combination of ataxia and early and severe autonomic failure is typical of the cerebellar type of MSA (MSA-C) [63]. Therefore, the clinical presentation of MSA-C may overlap that of SCAs, thus representing a relevant differential diagnosis in the absence of a clear family history. Furthermore, neuropathological studies in SCA2 revealed marked olivo-ponto-cerebellar atrophy pattern highly resembles that of MSA. SCA2 may even display pontine hyperintensities at MRI, the so-called hot-cross-bun sign, which is considered typical of MSA [18].

In patients with both autosomal dominant and recessive ataxias, various forms of dysautonomia have been described, but are generally less well documented [61, 62]. For example, a study of cardiac autonomic dysfunction (sympathetic / parasympathetic heart rate and blood pressure tests) in SCAs found some degree of abnormality in all 14 patients with SCA1, 2 and 3. Severe changes were found in more than half of their sample, being much more common in SCA3, followed by SCA1 [19]. The same group previously reported a predominantly parasympathetic autonomic cardiac dysfunction in approximately 70% of SCA1, 2 and 3 patients, with a strict correlation with disease duration in SCA1 [20]. The suggestion of cardiac denervation in SCA3 had been suggested in an earlier study using 123-labeled MIBG [21] and confirmed using different methodologies, including a pathological analysis showing reduction of intermediolateral column cell count in the spinal cord [22–24].

In SCA2, clinical manifestations of various forms of dysatonomia were also described in up to 95% of cases studied, including vasomotor disorders (orthostasis, Raynaud’s phenomenon), constipation, incontinence, tachycardia, and exocrine gland dysfunction [25]. Bladder and gastrointestinal symptoms as the most common autonomic features among SCA 2 patients [18], and severe cases with early onset also may occur [63]. On the other hand, SCA6 and SCA10 patients seem to have preserved autonomic function [15, 64]. Both SCA6 and SCA10 may present as pure spinocerebellar ataxias in some populations, however, Kim et al. [65] found 50% (4/8) SCA6 patients presenting dysautonomia (two with impotence, one with urinary frequency, and one with orthostatic dizziness). A single case report of SCA17 presenting as an MSA-C phenotype with dysautonomia has also been described [66].

#### Sleep disorders

Sleep disorders are widely recognized but paradoxically clinically overlooked NMS in the context of several neurodegenerative diseases, being an important modifiable factor in the quality of life of patients [67]. Among the subtypes of SCAs, SCA3 has the highest prevalence and range of sleep disorders throughout the phases of the disease [67]. The spectrum of sleep disorders described includes restless legs syndrome (RLS), REM sleep behavioral disorder (RBD), periodic movement of limbs during sleep (PMLS), sleep apnea, insomnia and excessive daytime somnolence (EDS) [68]. RBD has been particular and consistently described in up to 55% of SCA3 series [26, 27]. A clinical and questionnaire based study confirmed the higher frequency of RBD and RLS in SCA3. In this study, anxiety and depression were significantly correlated with RDB, but not with RLS [69]. Similar to PD, in SCA3, RBD is not only highly prevalent but may also precede the onset of motor symptoms [70].

SCA3 patients may also present frequent non-REM sleep parasomnias, including higher frequency of arousals from slow wave sleep, and confusional arousal/sleep terrors and nightmares [71]. Obstructive sleep apnea and corresponding increased daytime somnolence has been described in almost half of a sample of SCA3 patients [72]. In SCA2, studies show that most patients subjectively report good sleep quality and no RBD symptoms. Polysomnographic studies however, show REM without atonia in about half of patients, suggesting subclinical RBD, accompanied by reduction of REM density, a finding that was replicated on a larger cases control study [73, 74]. In SCA6, there is no evidence of increased prevalence of RLS, RBD or REM sleep without atonia, and clinical and physiological findings are more non specific and subtle, including increased daytime somnolence, worse subjective sleep quality, reduction in slow wave sleep and a higher frequencies of snoring and other respiratory disorders [75–77]. A similar relatively benign sleep pattern was described for SCA10 [78] while a study comparing SCA3, 10 and controls found a prevalence of RLS of almost 36% for RLS and 43% for RBD in SCA3 but findings similar to controls for SCA10 [15]. On the other hand, daytime sleepiness scores for SCA10 patients were elevated compared to controls, similar to SCA3 [15]. A single case report of respiratory stridor during sleep similar to that observed in MSA has been described recently in SCA17 [79].

Although the available literature is limited, RLS and periodic limbs movement of sleep (PLMS) have been described in SCAs, with a prevalence ranging from 20 to 30%, reaching up to 45% in patients with SCA3. One study of a mixed sample of 58 patients with SCA1, 2 and 3 found a frequency of 28% versus 10% in healthy controls. The occurrence of RLS was not related to age of onset disease, gender, CAG repeat length or findings of neurophysiological testing, but was positively correlated with age [67, 80]. In SCA6, only one small series of 5 patients analyzed the occurrence of RLS and motor activity during sleep, showing clinical signs of RLS in two and PLMS in all of them, although the impact on overall sleep quality was considered minor [81]. The previously mentioned study that analyzed NMS in SCA3, 10 and controls confirmed the higher prevalence of RLS in SCA3 (35.7%) but did not find it to be elevated in SCA10 [15]. Kapoor & Greenough [82] described a case of a SCA13 woman that presented with insomnia and RLS, and a polysomnogram with periodic limb movement index, obstructive sleep apnea and absence of REM sleep.

Excessive daytime sleepiness has been frequently found in patients with SCA3, however, the pathophysiologic mechanism involved remains uncertain. In addition, other sleep disorders have been reported, such as obstructive sleep apnea, insomnia, nocturia, and hypnagogic hallucinations [83].

#### Psychiatric symptoms

Neuropsychiatric symptoms may be part of the clinical spectrum of SCAs, however, their timing of occurrence along the disease course, prevalence, and how much they differ among the subtypes of SCA remain undetermined [28]. In terms of their spectrum, features of depression are by far the most common, however, studies also indicate that anxiety is a frequent complaint, especially among patients with SCA3 [29].

Cecchin et al. [30], for instance, detected depression in 33.5% of 79 SCA3 cases. McMurtray et al. [28] studied the frequency of depressive symptoms in 76 patients with SCA1, SCA2, SCA3 and SCA6 detecting significantly higher prevalence among subjects with SCA3 (60%), compared to other SCA subtypes [SCA1 (25%), SCA2 (23%), and SCA6 (27%)]. Klinke et al. [31] studied the severity of symptoms and did not detect scores indicating severe depression in a total of 32 patients with SCA1, 2, 3 and 6, but found mild depression more frequently among SCA1 and 6 cases.

A clinical study of SCA1 and SCA2 also found worse scores of depression and apathy between both groups compared to controls [32]. A study comparing SCA3, SCA10 and controls found a similarly higher prevalence of depression and anxiety for both SCAs subtypes compared to control [15].

A large study assessing self-rated health status in 526 genetically and clinically defined patients with SCA1, SCA2, SCA3 and SCA6 found a prevalence of 17.1% for depressive syndromes and 15.4% when assessed clinically in an interview in a subset of 26, which was equally distributed among all SCAs subtypes. The clinical profile of depression did not differ from a sample of patients with major depressive disorder. After logistic regression analysis, the independent predictive factors for depression were ataxia severity and female sex [33]. Saute et al. [53] performed magnetic resonance imaging (MRI) in 30 SCA3 patients and found no correlation between the measures of volumes of the brainstem, midbrain, pons and cerebellum, with depression. Braga-Neto et al. [84] studied 22 SCA3 patients with neuropsychological assessment and dopamine transporter (DAT) densities and found no significant correlation between DAT density and cognitive performance or Sniffin Sticks scores. The nature of depressive symptoms – whether organic or reactive – remains controversial. The connections of the cerebellum with the non-motor cortex and subcortical areas associated with the processing of emotions, suggest a role of this on the mood and personality of the patients [85]. However, despite its importance, impact and relative frequency, depression is probably underdiagnosed and consequently undertreated among SCA patients [33].

#### Cognitive symptoms

Cognitive disorders have been described in various forms of SCA. The presence of these manifestations, associated with the classical cerebellar signs of the motor syndrome, can be explained by the disconnection at various levels of the cerebro-cerebellar circuit, the so-called cerebellar cognitive affective syndrome (CCAS) [86]. The CCAS was characterized by Schmahmann & Sherman [87] based on a study of 20 patients with cerebellar disease using combined neuropsychological and neuroimaging assessments as follows: I) executive dysfunction (poor planning, abstract reasoning, etc.; II) visuospatial memory impairment; III) personality changes (affective swelling and disinhibition; IV) difficulties interpreting and producing logical sequences; V) language difficulties (mild anomia and agramatism).

Cognitive disorders may be present in several types of SCAs, although established dementia cases have been related to more restricted subtypes, as shown in Table 2. Bürk et al. [34] studied 36 SCA1, SCA2 and SCA3 patients, and a healthy control group detecting significant executive dysfunction in SCA1, as well as mild deficits in verbal memory among the whole sample of ataxic patients overall. Interestingly, the same group had already demonstrated that a quarter of their sample of SCA2 patients were demented, arguing that intellectual impairment represents an independent and significant part of the disease phenotype [35]. Trojano et al. [36] found a significant relation between clinical severity and verbal memory in 15 patients with SCA2, while Gambardella et al. [37] detected early and selective executive dysfunction in 17 SCA2 cases.

**Table 2:** Cognitive disturbances in the spinocerebellar ataxias

Cognitive disturbance	Spinocerebellar ataxia
Cognitive dysfunction	SCA1, 2, 3, 6, 7, 8, 10, 12, 17, 19, 21, DRPLA
Dementia (confirmed by specific testing)	SCA2, 8, 12, 17, 19, DRPLA
Mental retardation during childhood	SCA13

*SCA* Spinocerebellar ataxia, *DRPLA* Dentatorubropalidoluisian atrophy

In SCA3, a recent neuropsychological study of 15 Japanese cases showed that visual working memory, attention and inhibition control were preserved, while word retrieval, as part of semantic memory, was impaired [38]. A similar pattern was also detected in Brazilian and Chinese patients with SCA3, added by visuospatial dysfunction in the later [39, 40]. A previous longitudinal study showed progressive deterioration of memory and learning abilities, suggesting that cognitive dysfunction is an integral part of the SCA3 phenotype [41].

Kawai et al. [42] studied 13 patients with SCA6 using neuropsychological tests and brain SPECT demonstrating mild visual memory, verbal fluency and executive dysfunction that correlated with prefrontal hypoperfusion. When compared with SCA3, Garrard et al. [43] evidenced a cognitive function with poorer performance in the SCA3 compared to SCA6, with more prominent memory and executive function deficits. Another study of SCA6 patients did not find cognitive abnormalities compared to healthy controls [44]. A series of 19 African patients with SCA7 described dementia in almost half of affected cases, particularly as a late feature [45]. A longitudinal study, however, found only minor cognitive changes in SCA7, comparable to SCA6 [46]. Finally, the above mentioned study that compared SCA3, 10 and controls found only mild cognitive impairments in SCA 10, namely in terms of frontal and phonemic verbal fluency [15].

#### Olfactory function

Hyposmia has been well documented as an early and frequent sign in different neurodegenerative disorders, such as PD and Alzheimer disease (AD). The occurrence of this NMS among SCAs is not well established [88, 89].

Velazquez-Perez tested olfactory threshold, description, identification and discrimination in a large sample of SCA2 patients and healthy controls, finding significant abnormalities, in a pattern similar to that of PD and AD [47]. A similar assessment in SCA7 also found deficits in identification, however normal detection thresholds [48]. In SCA3, findings are not uniform: Braga-Neto et al. [49] found odor identification dysfunction in a controlled study with 41 SCA3 subjects.

On the other hand, two studies by Moscovich et al. [50, 51] analyzed different samples of relatively large numbers of patients with SCA10, SCA3, familial ataxia of unknown genetic basis PD, and healthy individuals, finding no significant difference in odor identification testing among the groups with SCAs compared to healthy controls. The study by Fernandez-Ruiz et al. [52] also did not find olfactory impairment in patients with SCA3 although it did so for SCA2, in line with the Velazquez-Perez study mentioned previously [47].

## Conclusion

Among the SCAs, the combinations of motor features and NMS of each type of SCA have been relatively well studied and serve as clues to guide clinical diagnosis and genetic testing. However, for the construction of new research protocols and advances in the research for the treatment of SCAs, it is necessary that other studies in this subject be made. The conjunction and integration of groups around the world who are studying SCAs is critical to this.

## Data Availability

Not applicable.

## Abbreviations

AD: Alzheimer disease
ADCA: Autosomal dominant cerebellar ataxia without identified gene mutation
CCAS: Cerebellar cognitive affective syndrome
DAT: Dopamine transporter
EDS: Excessive daytime somnolence
MRI: Magnetic resonance imaging
MSA: Multiple systems atrophy
NMS: Nonmotor symptoms
PD: Parkinson’s disease
PMLS: Periodic movement of limbs during sleep
RBD: REM sleep behavioral disorder
RLS: Restless legs syndrome
SCA: Spinocerebellar ataxia
